# Global survey of mobile DNA horizontal transfer in arthropods reveals Lepidoptera as a prime hotspot

**DOI:** 10.1371/journal.pgen.1007965

**Published:** 2019-02-01

**Authors:** Daphné Reiss, Gladys Mialdea, Vincent Miele, Damien M. de Vienne, Jean Peccoud, Clément Gilbert, Laurent Duret, Sylvain Charlat

**Affiliations:** 1 Laboratoire de Biométrie et Biologie Evolutive, Université Lyon 1, CNRS, UMR 5558, Villeurbanne, France; 2 Laboratoire d’Ecologie et Biologie des Interactions, Equipe Ecologie Evolution Symbiose, Université de Poitiers, CNRS, UMR 7267, Poitiers, France; 3 Laboratoire Evolution, Génomes, Comportement, Ecologie, CNRS Université Paris-Sud UMR 9191, IRD UMR 247, Gif sur Yvette, France; University of Wyoming, UNITED STATES

## Abstract

More than any other genome components, Transposable Elements (TEs) have the capacity to move across species barriers through Horizontal Transfer (HT), with substantial evolutionary consequences. Previous large-scale surveys, based on full-genomes comparisons, have revealed the transposition mode as an important predictor of HT rates variation across TE superfamilies. However, host biology could represent another major explanatory factor, one that needs to be investigated through extensive taxonomic sampling. Here we test this hypothesis using a field collection of 460 arthropod species from Tahiti and surrounding islands. Through targeted massive parallel sequencing, we uncover patterns of HT in three widely-distributed TE superfamilies with contrasted modes of transposition. In line with earlier findings, the DNA transposons under study (TC1-Mariner) were found to transfer horizontally at the highest frequency, closely followed by the LTR superfamily (Copia), in contrast with the non-LTR superfamily (Jockey), that mostly diversifies through vertical inheritance and persists longer within genomes. Strikingly, across all superfamilies, we observe a marked excess of HTs in Lepidoptera, an insect order that also commonly hosts baculoviruses, known for their ability to transport host TEs. These results turn the spotlight on baculoviruses as major potential vectors of TEs in arthropods, and further emphasize the importance of non-vertical TE inheritance in genome evolution.

## Author summary

Transposable elements are chunks of DNA that can produce copies of themselves. New copies usually insert in the genome of their carrier but are occasionally subject to horizontal transmission between organisms, sometimes belonging to evolutionarily-distant lineages. Previous surveys have established that the probability of such events is largely conditioned by the transposition mechanism. For example, elements with an RNA intermediate tend to be less frequently involved in horizontal transfers. Here we investigate host taxa as another potential explanatory factor of variation in horizontal transfer rates. Using targeted sequencing in hundreds of insects and other arthropod species collected in South Pacific islands, we found that butterflies and moths (Lepidoptera) show an abnormally elevated rate of horizontal transfers. Previous studies have established that Lepidoptera are also commonly attacked by baculoviruses, large viruses that can transport host DNA. Taken together, these findings point to baculoviruses as a major suspect for transposable elements transfers across arthropod species.

## Introduction

Metazoans are mostly sexual organisms, meaning that their reproduction is strictly associated with a process of massive genomic exchanges between two close relatives. However, they also occasionally acquire DNA from evolutionarily-distant lineages, which produces an important source of heritable variation and deeply affects genomes’ evolutionary dynamics [1–5]. In fact, it appears that the single most abundant component of eukaryotic genomes, transposable elements (TEs), derives in large part from recurrent HTs (from now one referred to as Horizontal TE Transfers, or HTTs) [6,7]. In arthropods, the most diverse animal phylum, a recent survey based on full genomes from public databases identified thousands of such events [8]. In addition to revealing the abundance of horizontally-acquired DNA within genomes, this study confirmed the previously suggested trend that variation in HTT rates can in large part be explained by the TE biology: DNA transposons tend to move more often than LTR-retroelements, which move more often than non-LTR retroelements [6,9,10].

Here we investigate the hypothesis that host taxa might represent an additional important predictor of HTT rates. To this end, instead of using whole-genome databases, subject to strong inherent taxonomic biases, we use a large and random field sample of arthropods to uncover patterns of HTT in three widespread TE superfamilies. This design allows us to assess variation and commonalities in patterns of HTT across TE superfamilies and across host taxa, based on species that actually co-occur in a geographically-delimited region.

In accordance with earlier reports, we found that the DNA transposons under study (Tc1-Mariner superfamily, hereafter “Mariner” for simplicity) are most often transferred, followed closely by LTR-retroelements (Copia superfamily). Non-LTR retroelements (Jockey superfamily) harbor the highest diversity, both in terms of molecular variation and host range, but a comparatively low rate of HTT. To assess variation across host taxa, we used simulations of random HTT scenarios constrained by the actual TE distribution and taxonomic composition of our sample. Strikingly, one pattern emerges that holds across the three TE categories: a large excess of HTTs in the Lepidoptera. The observed number of HTTs in this clade is much higher than expected from the global HTT rate, and most transfer events take place between Lepidoptera species. In combination with the earlier finding that baculoviruses, DNA viruses that are very prevalent in Lepidoptera, often carry host TEs [11,12], this finding points to baculoviruses as key drivers of HTT in arthropods.

## Results

### TEs diversity and distribution

**Fig 1:**
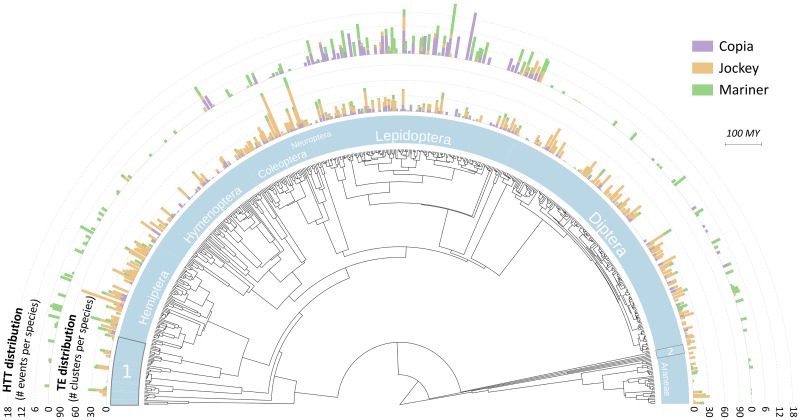
TE and HTT distributions in relation with host phylogenetic diversity. The tree is a chronogram based on Bailly-Bechet et al. [55]. Starting from the center, the first layer of data indicates the distribution of TEs (number of families per host species). The second layer indicates the estimated number of HTTs per species (mean number across 1000 plausible HTT scenarios). Boxes 1 and 2 represent arthropod orders with small sample size. Box 1 (from top to bottom): Pscocoptera (n = 10), Thysanoptera (n = 5), Orthoptera (n = 3), Mantodea (n = 1), Blatodea (n = 5), Isoptera (n = 1). Box 2 (from top to bottom): Amphipoda (n = 2), Scolopendromorpha (n = 1), Sarcoptiformes (n = 2), Ixodida (n = 1).

To investigate variation in HTT rates among arthropod taxa, we used degenerate PCR and amplicon sequencing on 460 species collected in Tahiti and surrounding islands. This sample spans 19 orders of arthropods, with a large majority of insects (Fig 1, S1 Table). It represents a random subset of a larger collection mainly obtained through sweeping nets and malaise traps, without particular taxonomic focus [13]. TE sequences were assigned to superfamilies based on their similarity with reference proteins [14] and clustered into families (> 80% nucleotide identity based on BLAST alignments) [15]. Only families occurring in two specimens within each species were retained. The data confirmed that although our approach relied on PCR, a large diversity was captured within the Copia, Jockey and Mariner superfamilies (Fig 1, S1 Table, S1 Text), making them suitable for investigating the host influence on HTT rates. On the contrary, Gypsy (LTR-retroelement) was found mostly restricted to Diptera, and comparisons with genome-based surveys [8] suggested we only captured a subset of the actual diversity of this superfamily. The Gypsy data was thus excluded from further analysis.

Jockey elements appear far more diverse than the others in terms of sequence diversity (Fig 1, Table 1) making 77% of all families, while Copia and Mariner represent only 9% and 14%, respectively. This partly stems from the wide distribution of Jockey, which is present in 252 host species (that is 55% of the surveyed sample) versus 174 and 129 for Copia and Mariner, respectively. Jockey is also more diverse within genomes, with 15 families per host species on average, versus 4 and 3.5 for Copia and Mariner, respectively.

**Table 1:** Minimal number of HTTs across superfamilies, in relation with their respective molecular diversities as measured by the total number of families.

Superfamily	Nb. of families	Min. nb. of HTTs	HTT rate (Nb. of HTT per TE family)
Copia	696	123	0.18
Jockey	3876	34	0.01
Mariner	453	175	0.39

### HTT rates variation across TE superfamilies

**Fig 2:**
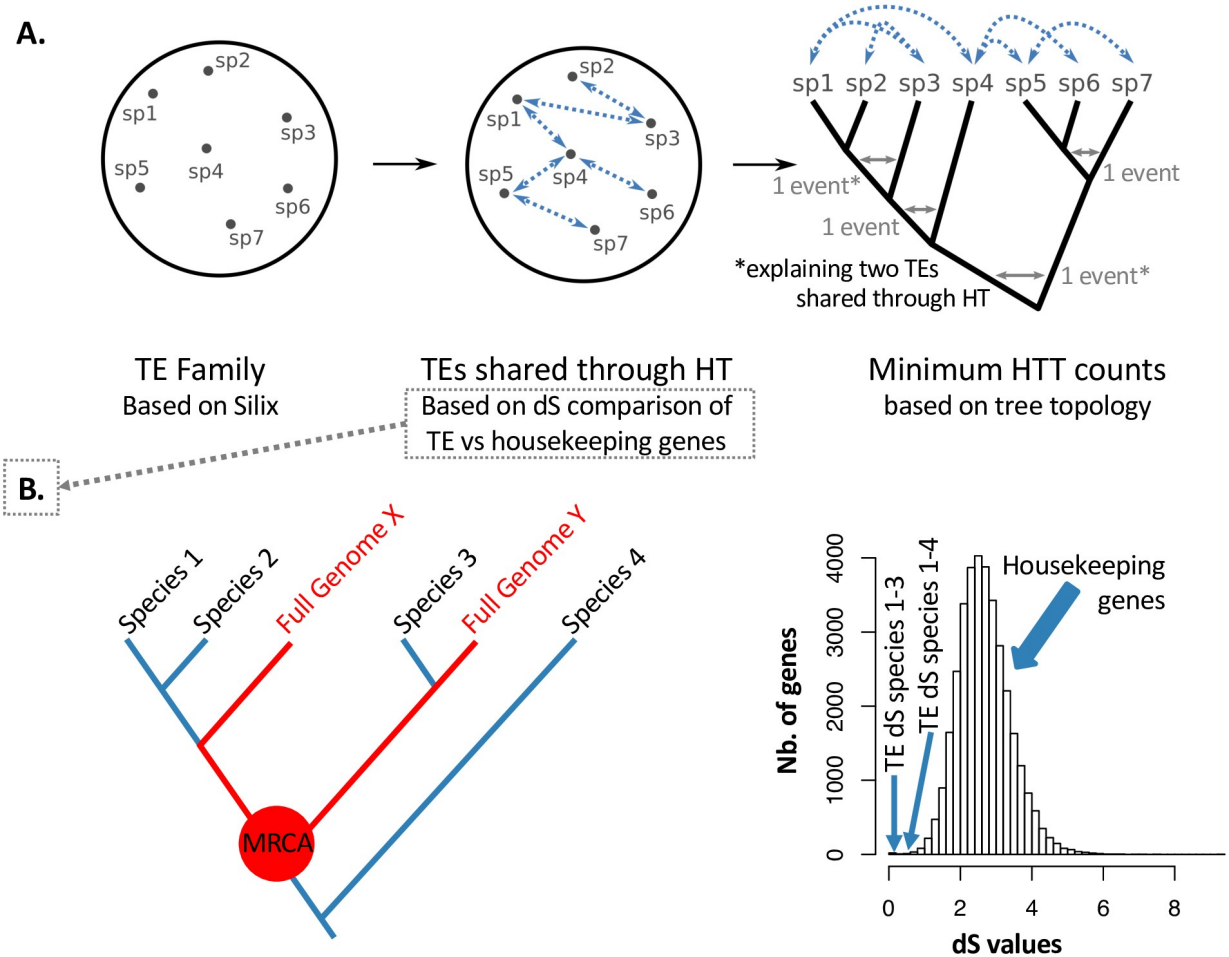
A graphical description of our HTT detection procedure. Panel A: a general overview. We first group into families highly similar TEs (>80% nucleotidic identity) occurring in different species. Following a previously proposed formalism [43], we then establish networks of species sharing horizontally transferred TEs, based on dS comparisons of TEs versus housekeeping genes. Finally, we use the host phylogeny to compute the minimal number of HTTs, assuming that any cases of TE shared through HT between two sister clades can be explained by a single HT event. Panel B: details of the second step. The identification of TE families shared through HT first implied computing a tree including all species from our sample (labeled in black) in addition to arthropod species for which a complete genome was available (labeled in red), using sequences of the mitochondrial gene CO1 with imposed topology for deep nodes [55] (S1 Fig). The Most Recent Common Ancestor (MRCA) of Species X and Y (red dot) is the same as that of species 2 and 3. In other words, the divergence time between species X and Y is the same as that between species 2 and 3. We can thus use the distribution of dS values observed between housekeeping genes in genomes X and Y (panel B) to test the null hypothesis that TEs shared between species 2 and 3 have been inherited vertically. On the contrary, we cannot test this hypothesis for TEs shared between species 1 and 2. The MRCA of species 2 and 4 is older than the MRCA of species X and Y; we can thus use the X-Y dS distribution to conservatively test the hypothesis that TEs between species 2 and 4 have been inherited vertically. In this toy example, species 1 and 3 and species 1 and 4 share TEs that have less than 5% chance to result from vertical inheritance. We thus infer two cases of TEs shared through HT.

To detect HTTs, we followed the procedure summarized in Fig 2. We first identified all TE families occurring in more than one species as HTT candidates. Following a previously proposed rationale [8,10,16,17] we then looked for TEs showing an abnormally low neutral divergence (dS values) in comparison to a genome-wide reference. To this end, we used arthropod full genomes to compute the distribution of dS values of housekeeping genes between any two species in our sample, and assessed if the observed TE dS was likely sampled from this distribution. Notably, HTT between closely-related species may be untestable with this approach due to the lack of reference genomes in their respective clades (e.g. species 1 and 2 in Fig 2b) (S1 Fig). However, these cases were rare: our dataset includes 2,692 pairs of species sharing TE families, and HTT could be tested in 2,407 of those (90% of the cases). In the vast majority (98%) the hypothesis of vertical inheritance was rejected at the 5% threshold risk, that is, the observed dS was smaller than the 5% quantile of housekeeping genes dS values. Having thus established a list of shared TE families through HT, we used host phylogeny to compute the minimal number of HTT events, taking into account the possibility that some events may have occurred before the split between extant species, thus explaining more than one case of shared TEs through HT (Fig 2a). In these situations, we approximated that HTT scenarios should involve only one randomly picked descendant of the ancestor lineage where the event actually took place.

Overall, at least 332 HTT events are required to explain our data (Fig 1, Table 1, S2 Table). As expected from the detection approach, synonymous divergence between transferred elements confirms that most of the inferred HTT events are recent, with a majority of dS values below 0.5 (S3 Fig). In 32 species, we retested the occurrence of highly similar TEs with PCR and/or Sanger sequencing. In all cases, these tests validated the amplicon sequencing data. Consistent with a previously established trend [6,8–10], we observe that Mariner elements transfer at the highest rate, with 52% of all events being associated with this superfamily, while 37% of the events involve Copia, and only 10% Jockey. Variation between superfamilies in abundance within host genomes (a metric that cannot be estimated with the present data) may explain part of this pattern. Nevertheless, taking molecular diversity into account (the number of families within each superfamily) reveals that more diverse elements are not necessarily more prone to HTT: Jockey elements are involved in only 10% of the HTT cases although they are by far the most diverse (Table 1). Thus, we observe on average 0.01 HTT event per Jockey family, but ~20 times more per Copia family, and ~40 times more per Mariner family.

### HTTs rates variation across taxa

We investigated the hypothesis that, in combination with TE biology, the nature of host taxa might contribute to explain HTT patterns. A visual inspection of the distribution of HTT events is highly suggestive of such variation (Fig 1, S3 Fig), with an excess of events in the Lepidoptera order (68% of the HTT cases). However, this order is also the most abundant in the initial sample, and more generally, the taxonomic composition and phylogenetic structure of the data may produce variation among taxa even under a random process. To compare HTT rates across taxa while taking possible sources of bias into account, we simulated a large number of random HTT scenarios constrained by the taxonomic and phylogenetic structure of our sample and by the observed number of HTT events. Specifically, for each superfamily, we randomly defined pairs of species sharing horizontally-transferred elements, picked among those carrying the element under study. We thus produced 1,000 random HTT scenarios.

**Fig 3:**
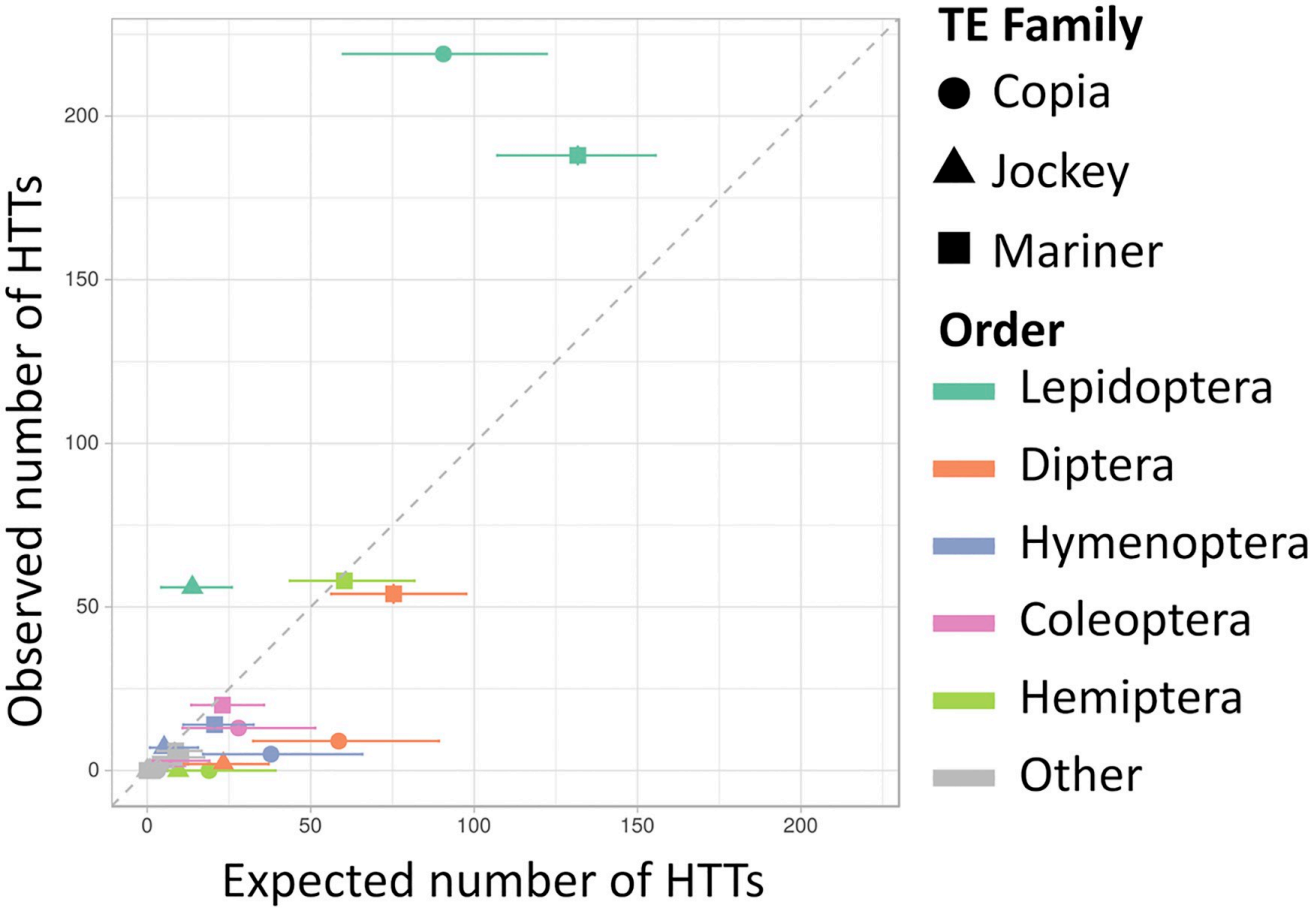
A comparison between the observed and simulated number of species involved in HTT in each arthropod order, and for each superfamily. Horizontal segments link the 2.5% and 97.5% quantiles of simulated values.

Comparisons between the observed and simulated HTT numbers across taxa and TE superfamilies are summarized in Fig 3. The most striking pattern is a large excess of observed HTT events in Lepidoptera for the three TE superfamilies as compared to the expectations. In the Copia superfamily, we observe 219 events in Lepidoptera when we expect 90 on average (between 75 and 115 in 95% of the simulations). In the Jockey superfamily, we observe 56 events in Lepidoptera, when we expect 13.7 on average (11.6–14.2). In the Mariner superfamily, we observe 188 events, when we expect 131.6 on average (117.5–149.7). In some orders comprising few analyzed species, the expected number of HTTs is close to 0, either due to low sample sizes or to low incidence of TE superfamilies (Fig 1). Aside from Lepidoptera, all arthropod orders with large sample size show comparatively reduced rates of HTT, with the observed number of events falling below or near the random expectations.

## Discussion

We surveyed the distribution and molecular diversity of three TE superfamilies in 460 arthropod species collected in Tahiti and surrounding islands. In contrast with previous analyses based on full genome databases [8], this taxonomically diverse dataset makes it possible to test the hypothesis that, in addition to the TE transposition mode, host biology could represent an important predictor of variation in HTT rates. Copia, Jockey and Mariner elements were found to be widely distributed across arthropod orders, making them suitable for our purpose. Although these elements show contrasted rates of horizontal transmission, in line with earlier reports, they follow a strikingly concordant pattern when it comes to inter-taxa variation: all three superfamilies show a large excess of HTTs in Lepidoptera.

Since the first reports of HTT in *Drosophila* flies, a number of methods have been proposed to detect HTT from genomic data. Following earlier studies [10,16] we compared here the synonymous divergence (dS) of TEs occurring in different species to a reference dS distribution, expected under vertical inheritance, computed with housekeeping genes from full genomes of close relatives. Our approach was conservative in several respects. First, any HTT instance was confirmed by two specimens from the same species, allowing us to rule out contamination, in combination with specific PCRs and re-sequencing experiments. The use of housekeeping genes to compute a null dS distribution is also conservative in the sense that such genes are more prone to codon usage selection and may thus show slower synonymous evolution than TEs [18]. Finally, as illustrated in Fig 2b, the reference dS distribution can correspond to Most Recent Common Ancestors (MRCAs) that are younger, but never older, than the MRCA of the two species under consideration.

Previous studies have suggested that the biology of TEs, and more specifically their transposition mode, might constitute a strong predictor of HTT rate [6,8–10,19–22]. Specifically, it has been reported that DNA transposons move more often than others between species, and LTR-retrotransposons more often than non-LTR. Although the present study focuses on only one superfamily within each category, it brings support to this trend, with 52% of all events being associated with Mariner, 37% with Copia, and only 10% with Jockey. Taking into account the diversity of each superfamily provides an even more contrasted picture: Jockey is involved in only a handful of transfers but is at the same time the most widely distributed, and the most diverse within genomes, with 15 families per host genome on average against 4 and 3.5 for Copia and Mariner, respectively. This means that elements of the Mariner and Copia superfamilies are respectively transferred 40 and 20 times more often than elements of the Jockey superfamilies.

Several hypotheses have been proposed to explain the propensity of DNA transposons to transfer horizontally [6,9,10,19,20]. First, it has been argued that the high stability of DNA, as compared to RNA, could facilitate the HT of DNA transposons, and to a lesser extent of LTR-retrotransposons, the cycle of which also includes a free DNA phase. Second, the reliance of DNA elements’ transposition on host factors appears much weaker than that of retrotransposons. For example, *in vitro* transposition of Mariner transposons can be achieved solely in the presence of a transposase [23–26] while retrotransposition is a more complex reaction, seemingly involving many interactions between host and TE factors [27–30].

The HT rate of the different TE classes should also be interpreted in the light of their long-term fate [19,20]. Our data indicates that Jockey elements are by far more diverse within genomes than Copia and Mariner, fitting the view that non-LTR elements may be longer-lived than others. Several factors have been identified that may contribute to this pattern [20]. First, the “replicative” transposition mode and *cis*-preference [31] of non-LTR elements, two features that decrease the negative impact of non-autonomous copies on the persistence of active ones. Second, non-LTR elements do not excise from their insertion site, in contrast with DNA elements (where excision is an integral part of the transposition process) and LTR-elements (where the Long Terminal Repeats provide a favorable terrain for deletion through unequal recombination). Regardless of the underlying explanation, the overall lower extinction rate of non-LTR elements may allow for long term coevolution with the host, resulting in intricate interactions with cells and reduced fitness impact [32]. Such high level of host specificity may often be incompatible with the ability to transpose in distant hosts [6].

Our study revealed large variation among arthropod orders in HTT rates, with a notable excess of events in Lepidoptera. While the earlier survey of HTT patterns by Peccoud et al [8] followed a different sampling scheme and analysis, a simple visual inspection suggests that such a trend was also present in this dataset, at least for retroelements (see figure 1 in [8]). Various aspects of host biology may underlie variation in HTT rates among taxa, such as differences in TE regulation systems, permeability of the germline, or exposure to HTT vectors [7]. Phylogenetic structure of the host taxa may also affect HTT rates if, as previously suggested, HTTs occur preferentially between close relatives [7,8,16,33]. Which of these hypotheses could explain the excess of HTTs in Lepidoptera documented here? At present, TE regulation in Lepidoptera appears to rely on piRNA as it does in other arthropods [34] and shows no signs of specificity that would facilitate the entrance and establishment of a horizontally transferred TE. We also have no indication that the germline would be more permeable in Lepidoptera than other insects. Phylogenetic structure does not appear either as a likely explanation of the observed pattern. Although HTTs tend to occur more frequently between close relatives in our data (S4 Fig), this effect is only driven by the excess of transfers within Lepidoptera (S5 Fig), while other groups including many close relatives, such as Diptera, show low rates of HTT (Fig 1).

Several lines of evidence suggest the vector hypothesis is more likely correct in the present case and point more specifically to baculoviruses as a potential explanatory factor. These large DNA viruses are very commonly found in Lepidoptera, and are rare in other clades [35–37]. Following a number of reports of TE insertions in baculoviruses [38–42], recent surveys based on deep sequencing of viral genomes have revealed an abundant presence of host TEs, some of which match documented cases of HTT [11,12]. Based on these findings, the high potential of baculoviruses as vectors of HTT in Lepidoptera has been previously emphasized [3]. Some authors have even explicitly suggested that considering the high prevalence of baculoviruses in Lepidoptera, an elevated HTT rate in that clade would provide support for their implication [20] (page 271). Our results are compatible with this conjecture, providing an independent line of evidence that baculoviruses may represent a major component of TE flux across species.

While the elevated rate of HTT in Lepidoptera constitutes the main outcome of our analysis, it should not minimize the more general observation, consistent with previous findings [8], that virtually all arthropod clades can be involved in the transfer of all TE categories, often between distantly related species. This means that in any case, baculoviruses represent only one among many possible vectors of HTTs, not only in Lepidoptera, where their main contribution is plausible but yet unproven, but also in other clades. Unravelling the various HTT routes and their respective contributions will require testing correlations between patterns of HTT and independent ecological data, providing information on which species are connected in nature and by which means [43] as well as direct experimental identification of the molecular mechanisms involved.

## Materials and methods

### Sample

The specimens used here are part of the SymbioCode sample, described in details in Ramage et al. [13]. In brief, terrestrial arthropods were collected in 2007 in four islands of the Society archipelago, French Polynesia: Tahiti, Moorea, Huahine and Raiatea. DNA extracts from 920 specimens were used in the present study, making 460 Operational Taxonomic Units (OTUs), that is, species-like groups as defined by mitochondrial sequence similarity (generally higher than 97%). OTUs were randomly chosen, on the sole criteria that they should include at least two specimens. Paired specimens were indeed used to validate each HTT through two independent replicates. As detailed in S1 Table, the specimens belong to 14 orders of insects, 3 orders of arachnids, one order of centipedes and one of malacostracans. Additional metadata and mitochondrial sequences are available in GENBANK and in the BOLD database (http://dx.doi.org/10.5883/DS-SYMC) (see S1 Table for accession numbers and links to specimen URLs).

### Degenerate primers design

Degenerate primers were designed to amplify four TE superfamilies: Copia (LTR-retrotransposon), Gypsy (LTR-retrotransposon), Jockey (non-LTR-retrotransposon) and Mariner (DNA transposon). To retrieve a broad diversity of sequences from these elements, the Copia, Gypsy and Jockey pol “canonical” proteins from the *Drosophila melanogaster* genome and the Mariner transposase protein from the *D*. *yakuba* genome were used as TBLASTN queries against the non-redundant protein database and whole genome shotgun databases of NCBI [14]. Retrieved sequences are listed in S3 Table. Protein sequences were aligned and degenerate PCR primers matching the most conserved regions were designed manually (S4 Table and S1 Data). In order to reduce degeneracy, two Jockey forward primers, and two Mariner forward and reverse primers were designed and used in equimolar amounts in the PCR mix. The expected amplicon sizes of *D*. *melanogaster* and *D*. *yakuba* reference sequences were 216 nucleotides for Copia, 696 for Gypsy, 402 for Jockey and 501 for Mariner.

### Amplification of TE superfamilies

All PCR primers were phosphorylated in their 5’ end to allow subsequent ligation of barcoding adapters. Two μl of template DNA were used with 0.1 mM of dNTPs and 0.5 U of Platinum Taq (Invitrogen) in a 25 μl final volume. For Gypsy and Mariner, 1.5 mM of MgCl2 were used whereas for Copia and Jockey MgCl_2_ concentration was increased to 2.5 mM. PCR was performed under the following conditions: 94 °C for 2 min, followed by 31 cycles of 94 °C for 30 s, variable annealing temperatures depending on the primers (S4 Table) for 30 s and elongation at 72 °C for 30 s (for Copia and Jockey) or for 45 s (for Gypsy and Mariner). A final elongation step of 5 min was included in all reactions. PCRs were performed in ten 96-well plates (92 specimens per plate plus 4 controls).

### Specimen pools

Amplicons of different TE superfamilies from the same specimen were pooled, since these can be separated following sequencing based on PCR primer sequences. Prior to pooling, amplicon concentration was adjusted to obtain relatively even sequencing depth across amplicons. To this end, amplicons were submitted to electrophoresis on GelRed stained, 1% agarose gel. Depending on visually-estimated band intensity 1, 2, 5 or 10 μL of PCR product were pooled.

### Barcoding adapters preparation

Barcoding adapters were used to assign reads to specimens following the multiplexed sequencing reaction. Adapters were designed and self-hybridized as in Meyer et al [44]. Briefly, a barcode adapter consists in an 8-nt molecular identifier (MID) followed by an SrfI digestion site (GCCCGGGC), followed by the reverse complement of the same 8-nt MID. In our protocol, a 3’ T was added to hybridize with the 3’ A overhangs of the PCR amplicons. Tags were designed using the barcrawl program with default options [45]. Ten μM of self-hybridized adapters were 5’ phosphorylated using 20 U of T4 Polynucleotide Kinase (Fremantas) in 1x of T4 DNA Ligase Buffer (Fermantas) in a final volume of 20 μl and incubated at 37°C for an hour. Kinase was then inactivated at 70°C for 10 min.

### Barcode ligation and pooling per plate

Specimen pools were ligated with 20 μl of 5’ phosphorylated barcode adapters using 5% PEG-4000 and 0.125 U/μl of T4 Ligase (Fremantas) in a final volume of 76 μl [44] and incubated at 4°C overnight. Ligase inactivation was performed at 70°C for 10 min. Reactions from the same PCR plate (10 plates, each containing 92 specimen pools) were pooled using 35 μl per reaction, to constitute 10 “plate pools”. Amplicons from each plate pool were purified using Macherey-Nagel NucleoSpin Extract II columns and eluted in 100 μl of H_2_O. Finally, an adapter fill-in step was performed as in Meyer et al. [44] followed by a purification in Macherey columns and elution in 50 μl of H_2_O.

### Dephosphorylation and digestion of barcoding adapters

Quantification of DNA for each plate-pool was performed with a NanoDrop spectrophotometer and equal DNA amounts were mixed together in the final pool to be sequenced. Dephosphorylation and restriction digestion by SrfI of the final pool was performed as in Meyer et al. [44], followed by a final purification in a NucleoSpin Extract II column. Finally, 50 μl of DNA (150 ng/μl) were sequenced by Macrogen in one eighth followed by one full GS-FLX Titanium run.

### Demultiplexing

454 reads were demultiplexed based on the barcodes using a Python program (available in folder 01 of the data repository) building on the approximate regular expression matching library TRE (https://github.com/laurikari/tre/) [46]. We tolerated up to two mismatches in the primer regions, but no mismatch in the MIDs. We thus searched and trimmed the adapters, including the PCR primers, and discarded reads that could not be unambiguously assigned to a specimen or a TE superfamily (script available in folder 02 of the data repository). After trimming, we applied a two-step procedure to extract sequences corresponding to the investigated TE superfamilies. First, sequences were compared with BLASTX to our reference TE-proteins (S1 Data) in order to select those that matched the expected TE superfamily (based on the presence of the associated primers in the sequence) with an e-value lower than 10^−7^. Second, the remaining sequences were compared with BLASTN to those that were selected at the first step (that is, to four enriched databases representing the four TE superfamilies) with a specific e-value threshold for each TE superfamily: 10^−100^ for Jockey, 10^−150^ for Mariner and Gypsy, 10^−200^ for Copia. These thresholds were set such that no sequence matched more than one TE-superfamily. Sequences were thus assigned to one of the four TE-superfamilies, or discarded.

To remove redundancy in the dataset, we aimed to assemble highly similar reads into consensus sequences. To this end, we used the Newbler transcriptome assembler [47], with the rationale that variations among closely related TEs within genome would generally mimic alternative splicing. A sequence was added to an isotig if it was at least 99% identical to another sequence of this isotig, with an overlap of at least 50-bp. The consensus sequences as well as non-assembled sequences (singletons) were used for further analysis. At this stage, some sequences were still found to be flanked by adapters, suggesting that several adapters had occasionally ligated to the same amplicon, although this was experimentally unexpected. To remove these regions, we compared our sequences to the reference proteins using BLASTX, and excluded any region that was not included in the blast alignment. This step also allowed us to orient sequences and determine their reading frame. All further analysis (dS estimations and clustering) were performed on the isotig consensus of assembled reads as well as singletons, excluding sequences that were shorter than 100bp. Raw sequences, as well as demultiplexed, trimmed and blast validated sequences, are provided in folder 03 of the data repository.

### Clustering of TEs into families

We used BLASTN to cluster sequences into groups of close relatives (families). To do this, all sequences of the same superfamily were blasted against themselves, and clustered via single linkage using the SiLiX program [15] (outputs of the SiLix clustering are provided in folder 03 of the data repository). Two sequences were clustered if they overlapped on at least 80% of the shortest sequence and showed at least 80% identity. In each family, species represented by less than two specimens were excluded. We verified that this dataset was devoid of experimental contaminants: no reads were observed from unexpected specimens, that is, specimens that had not produced a PCR amplicon for a given TE superfamily.

### Detection of horizontally transferred TEs

To detect horizontally transferred elements in our data, we looked for TEs showing higher levels of sequence similarity than expected based the divergence of their respective hosts species. This required comparing TE divergence to a null distribution, which should ideally be the neutral divergence of sequences that these species inherited from their most recent common ancestor (MRCA). Since we lacked such data from most sampled species, we used measures of synonymous divergence (dS distributions), previously obtained through the comparisons of 195 insect genomes at many core genes [8]. Specifically, a dS distribution was established for every MRCA (tree node) of the 195-species phylogeny, using pairwise dS values between the two lineages diverging at the corresponding node, as detailed in [8]. In order to select the distributions that were relevant to our study, we aimed at building a phylogenetic tree grouping these 195 species (hereafter called “full genome species”) and those from our own sample, based on the CO1 gene, that was obtained previously for our sample [13]. We could retrieve the CO1 sequence for 163 of the 195 full-genome species, using data from BOLD as well as BLASTN searches of a reference Lepidoptera sequence (GENBANK KX053497) against the full genomes (S5 Table). All retrieved CO1 protein sequences were aligned in Seaview using default settings [48] and a maximum likelihood phylogeny was computed in RaxML [49] under the GTRGAMMA model, constraining the relationships between arthropod orders from the topology of Regier et al [50]. Poorly supported nodes (with SH scores below 0.5) were collapsed in TreeGraph 2 [51]. An R script (available in folder 04 of the data repository) relying on the ape library [52] was then used to identify, on the basis of this phylogeny, which previously established dS distributions [8] could be used as a reference for a given species pair from our own sample, with the rational outlined in Fig 2b.

To estimate synonymous distances between sequences of TEs, we first aligned all sequences from a family using MACSE [53], which can align pseudogenes to a set of coding sequences (represented in our case by the initial set of reference sequences). Alignments are provided in folder 03 of the data repository. Using each multiple alignment containing at least two species, we computed a maximum likelihood tree using FastTree [54]. We then used an R script (available in folder 05 of the data repository) relying of the ape library [52] to remove redundancy from these trees, that is, to collapse any clade containing sequences from only one specimen into a single branch, selecting the longest sequence in this clade. We then extracted a sub-alignment from the complete MACSE multiple alignment, from which we computed the minimal dS value for all pairs of species using the same method as Peccoud et al. [8]. Cases of horizontally transferred TEs were inferred if the TE dS was lower than the 5% quantile of the reference genome distribution.

### Estimating the minimal number of HTs

Based on the list of shared TEs through HTs, inferred as described above, we estimated the minimal number of HTT events in our dataset, taking into account the possibility that a single transfer may be sufficient to explain several cases of shared TEs through HTs, if they occurred in the ancestor of recently diverged species (Fig 2a). Technically, we used an R script (available in folder 06 of the data repository) relying of the ape library [52] to extract (from the global CO1 host tree) the subtree of hosts species sharing a given TE family. We then considered all nodes in this tree and counted one HTT event if the two descending clades were connected through at least one pair of species sharing horizontally transferred TEs. Strictly speaking, we infer in these cases that HTT took place between ancestral branches, but for simplicity, we approximated that such events involved two extant species of our sample, randomly picked among those potentially involved. This procedure was repeated 1,000 times to estimate an average number of transfers per species per HTT scenario.

### Observed and simulated HTT networks

To produce null distributions for hypothesis testing, we simulated random HTT scenarios in R (scripts available in folder 07 of the data repository). For each TE superfamily, a simulated set consisted in randomly picking species (among those carrying the element under study) to relabel those involved in the observed network of species sharing horizontally transferred elements. This procedure maintains the distribution of the number of HTTs per species, as well as the structure of the HTT network. We however avoided having two species connected through HTT when these species could not, by construction, be connected in the real data (that is, species pairs in which we lacked reference genomes to compute the expected genomic dS). In Copia and Jockey superfamilies, this was simply achieved by randomly picking new species labels until no such forbidden pair was observed. For Mariner, this procedure could not completely avoid creating forbidden pairs because of the high number of transfers. Thus, within each simulated network where forbidden pairs occurred, we replaced each forbidden link by a link between initially unconnected species pairs, picked at random among “authorized” pairs in the network. This means that we maintained the total number of HTTs, but in a slightly different network configuration. For each TE superfamily, we thus produced 1,000 simulated datasets and analyzed each to count HTT events as we did for the real dataset (Fig 2).

The phylogenetic structure of the data led to more HTT events being inferred from simulated datasets than from the real one. In other words, the pattern shown in Fig 2a (where a single HTT event explains more than one case of shared TEs through HT), occurs less often in the simulated than observed scenarios. This difference was moderate, but varied in intensity between superfamilies, with 11% more simulated HT events for Copia, 16% for Jockey, and 26% for Mariner. To account for this bias in the comparisons of HT rates across superfamilies, we normalized the number of event per taxa, imposing the same total number of events in the simulated scenarios as in the observed scenarios.

### Bench validation of horizontal transfers

To test for DNA contamination mistakenly interpreted as HTT, 18 TE families for which we suspected HTT, representing 32 species pairs, were randomly picked and inspected experimentally by specific PCR. In 12 species pairs, the HTT inference was further confirmed by cloning and Sanger sequencing. Specific primers were designed according to the consensus of all sequences from a family (S6 Table). For 21 out of the 32 species pairs, we used as PCR template DNA from additional specimens, closely related to those analyzed (0% divergence based on the mitochondrial CO1 locus). For the remaining pairs, the putatively transferred element was amplified from the specimens used in the original 454 sequencing. Amplicons were cloned using the TOPO-TA cloning kit for Sequencing (Invitrogen) and sequenced by Genoscreen.

## Supporting information

S1 Fig A phylogenetic tree including our specimens (in black) together with species for which a complete genome was available (in red). Our specimens are labelled according to the following code: species and specimen ids (cf. S1 Table), followed by 5 letters indicating the order. Species with full genomes are labelled by full species names, followed by 5 letters indicating the order. The tree was computed in RaxML [49] with the mitochondrial gene CO1, using the GTRGAMMA model and constraining the relationships between arthropod orders from the topology of Regier et al [50]. Poorly supported nodes (with SH scores below 0.5) were collapsed in TreeGraph 2 [51].
(PDF)

S2 Fig Distribution of minimal synonymous divergence between horizontally transferred elements. The distribution is based on 2364 species pairs harboring abnormally similar TEs in comparison with whole genome divergence.
(JPG)

S3 Fig Visualization of observed and simulated HTTs on phylogenetic trees. All trees are the same, and links indicate HTT events. The level of transparency of links is proportional to the number of times a particular event is observed across 1000 HTT scenarios. On the left panel, HTT scenarios were sampled in relation with the observed network of shared TEs through HT (as illustrated in Fig 2). The right panel is based on 1000 random scenarios, sampled in relation with the distribution of TE superfamilies. Large orders are labelled at their root with the following code: 1 (Araneae), 2 (Diptera), 3 (Lepidoptera), 4 (Coleoptera), 5 (Hymenoptera), 6 (Hemiptera), 7 (Psocoptera + Thysanoptera).
(JPG)

S4 Fig Relation between phylogenetic distance between potential hosts and probability of HTs. Each circle represents the ratio of observed to expected HTT events in a window of 25 million years. The size of the circle is proportional to the expected number of HTs in this window. For example, for Copia, we observe about 2.5 more events in the 25–50 my window than would be expected. Each red line represents a linear logistic regression, computed from one simulated HT scenario, to evaluate the effect of genetic distance on the observed / expected ratio. Regressions were performed on log transformed ratio but are shown in linear scale. In each superfamily, the observed / expected HTs ratio decreases with genetic distance, but the effect is stronger for Copia and Jockey than for Mariner.
(JPG)

S5 Fig The effect of removing Lepidoptera on the relation between phylogenetic distance between potential hosts and probability of HTs. The legend is equivalent to that of S4 Fig, except Lepidoptera have been removed from the data, as well as all HTTs involving species from this order. The figure shows that the correlations between phylogenetic distance and HTT probabibilty are low or non significant once Lepidoptera are removed. This indicates that the distance effect in our data is mostly driven by the excess of HTTs within and among Lepidoptera species.
(JPG)

S1 Table A complete description of the specimens used together with results of the PCR screening and sequencing experiments. Specimen IDs correspond to internal SymbioCode IDs [13]. Headers are self-explanatory, except: “OTU” (Operational Taxonomic Unit, a unique identifier for the 460 species used); “pcr_copia”: indicates if an amplicon was obtained for Copia (and similarly for other superfamilies), numbers indicate amplicon intensity; “sequenced?”: indicates if at least one amplicon from this specimen was included in the sequencing experiment. “nb_reads_copia”: number of reads assigned to the Copia superfamily (and similarly for other superfamilies), following the elimination of reads from species represented by only one specimen in a given family.
(XLSX)

S2 Table Distribution of TE superfamilies and HTTs across species. Taxonomic details are redundant with S1 Table, but provided for information. “families_copia” indicates the number of Copia families obtained (and similarly for other superfamilies). “HTTs_copia” indicate the mean number of HTTs inferred for this species across 1000 scenarios compatible with the data (and similarly for other superfamilies).
(XLSX)

S3 Table Host species and accession numbers of the reference sequences. (XLSX)

S4 Table Degenerate primers. (XLSX)

S5 Table 163 CO1 sequences retrieved from the 195 species used by Peccoud et al [8]. CO1 sequences were either retrieved by species name search from the BOLD database, or by blast search on full genome data.
(XLSX)

S6 Table Primers used for the bench validation of HTs. (XLSX)

S7 Table Summary statistics of the sequencing data. (XLSX)

S1 Data Nucleotidic alignments of the reference proteins for each TE superfamily, together with degenerate primers. (ZIP)

S1 Text Further details, not essential to the main message of the paper, on the screening and sequencing results. (DOCX)
