# Supplementary figures and images for: Global survey of mobile DNA horizontal transfer in arthropods reveals Lepidoptera as a prime hotspot
Source: PLoS Genet. 2019 Feb 1;15(2):e1007965. doi: 10.1371/journal.pgen.1007965 (PMC6373975; doi:10.1371/journal.pgen.1007965)

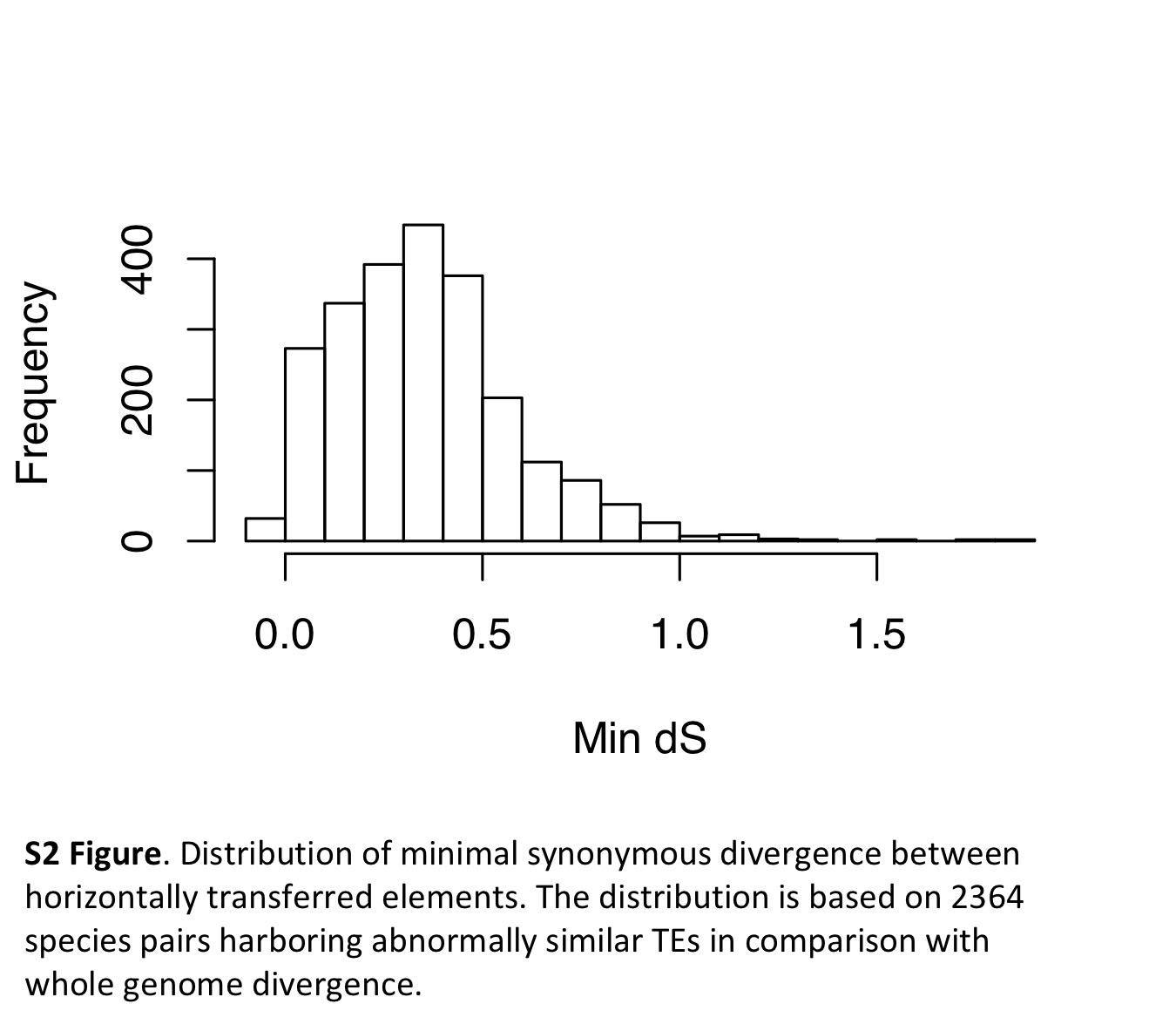

Supplement: S2 Fig — The distribution is based on 2364 species pairs harboring abnormally similar TEs in comparison with whole genome divergence. (JPG) [file pgen.1007965.s002.jpg]

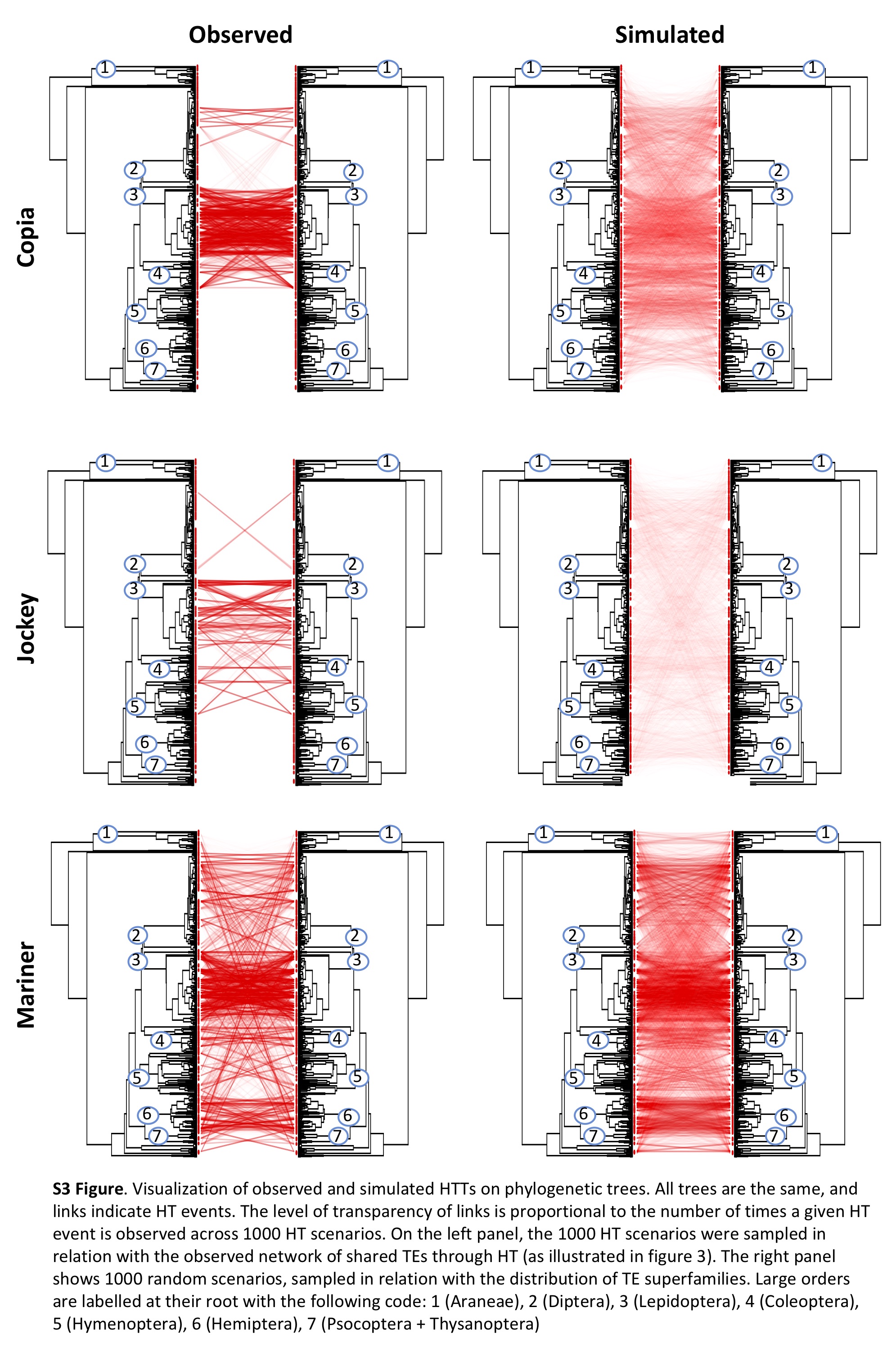

Supplement: S3 Fig — All trees are the same, and links indicate HTT events. The level of transparency of links is proportional to the number of times a particular event is observed across 1000 HTT scenarios. On the left panel, HTT scenarios were sampled in relation with the observed network of shared TEs through HT (as illustrated in Fig 2). The right panel is based on 1000 random scenarios, sampled in relation with the distribution of TE superfamilies. Large orders are labelled at their root with the following code: 1 (Araneae), 2 (Diptera), 3 (Lepidoptera), 4 (Coleoptera), 5 (Hymenoptera), 6 (Hemiptera), 7 (Psocoptera + Thysanoptera). (JPG) [file pgen.1007965.s003.jpg]

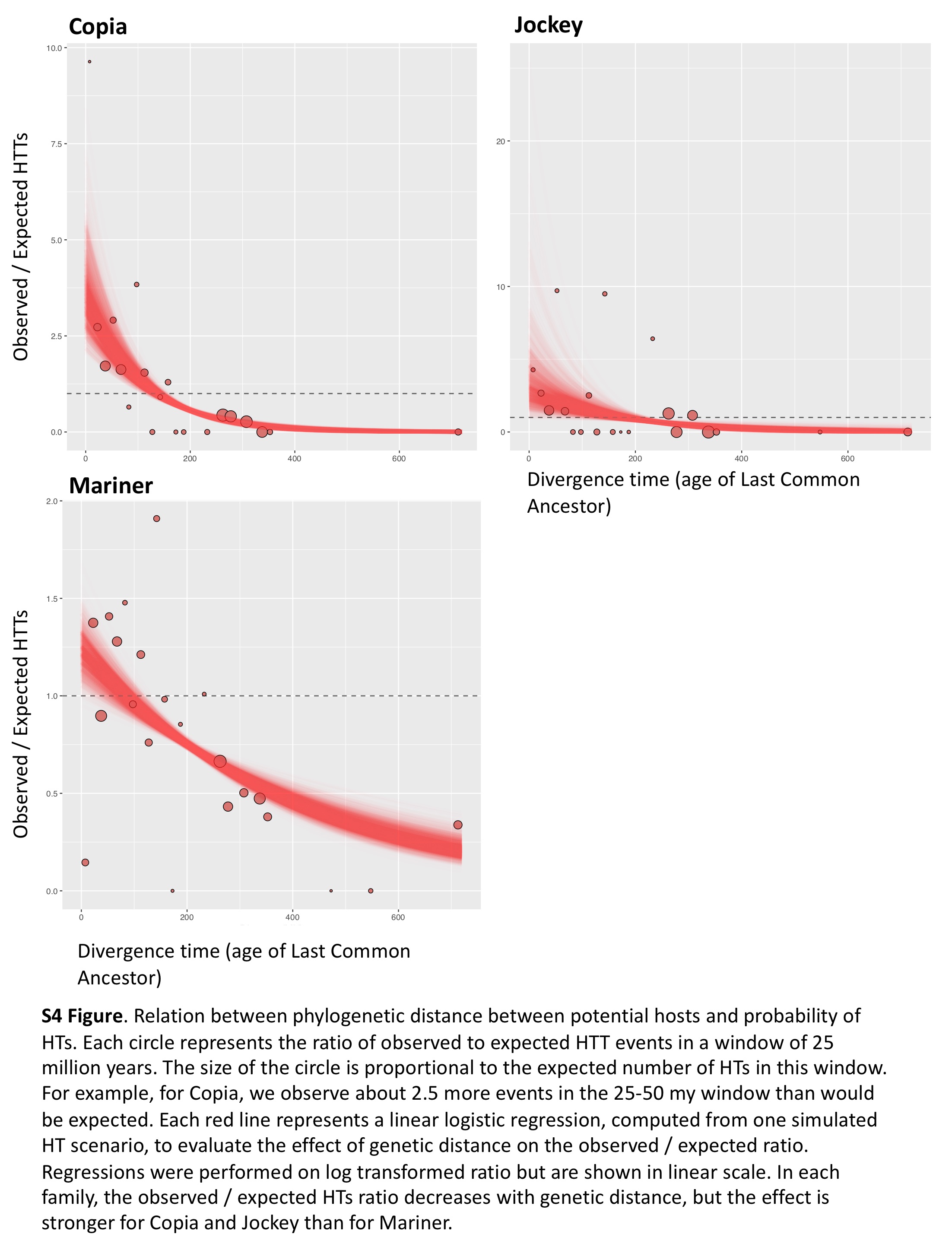

Supplement: S4 Fig — Each circle represents the ratio of observed to expected HTT events in a window of 25 million years. The size of the circle is proportional to the expected number of HTs in this window. For example, for Copia, we observe about 2.5 more events in the 25–50 my window than would be expected. Each red line represents a linear logistic regression, computed from one simulated HT scenario, to evaluate the effect of genetic distance on the observed / expected ratio. Regressions were performed on log transformed ratio but are shown in linear scale. In each superfamily, the observed / expected HTs ratio decreases with genetic distance, but the effect is stronger for Copia and Jockey than for Mariner. (JPG) [file pgen.1007965.s004.jpg]

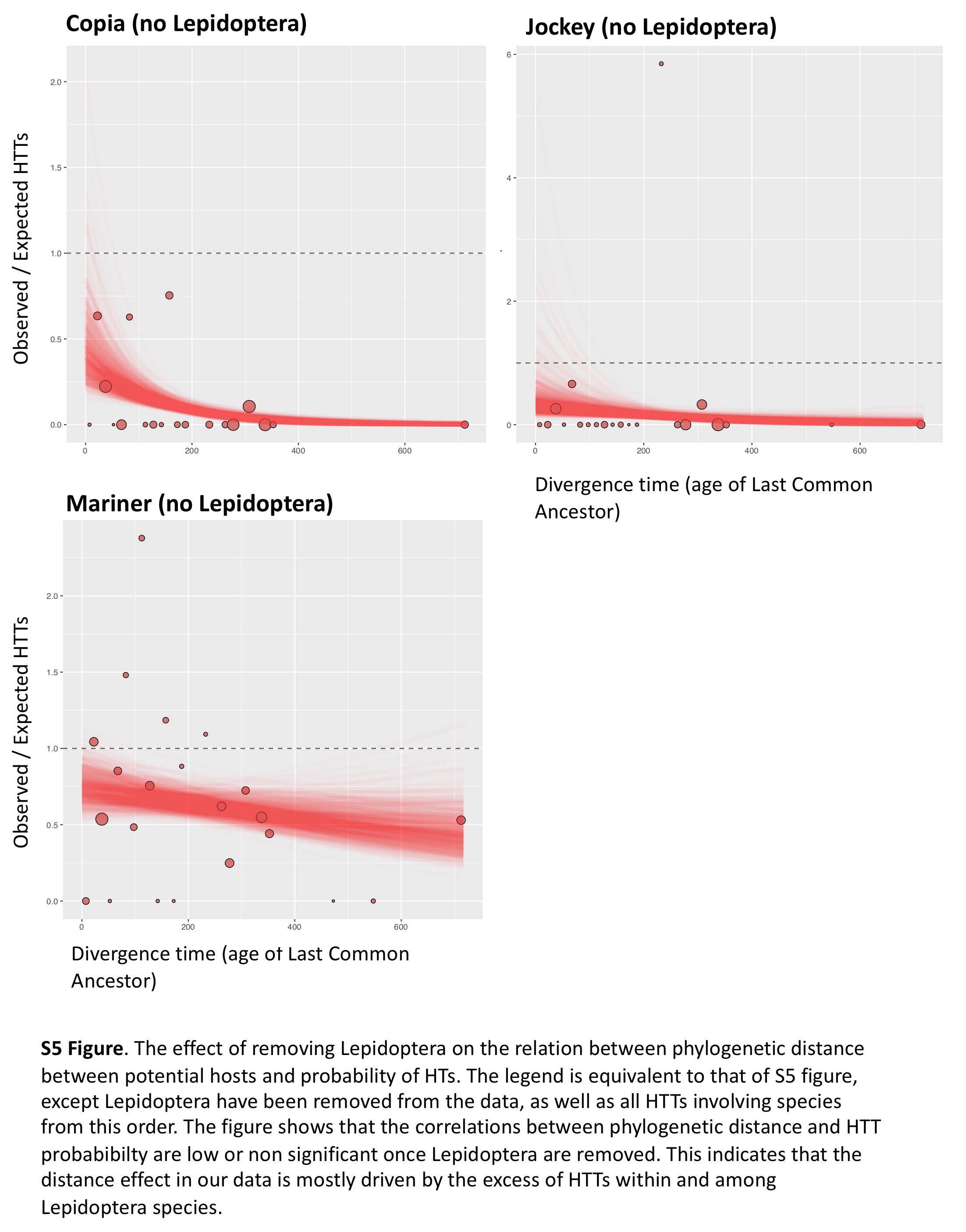

Supplement: S5 Fig — The legend is equivalent to that of S4 Fig, except Lepidoptera have been removed from the data, as well as all HTTs involving species from this order. The figure shows that the correlations between phylogenetic distance and HTT probabibilty are low or non significant once Lepidoptera are removed. This indicates that the distance effect in our data is mostly driven by the excess of HTTs within and among Lepidoptera species. (JPG) [file pgen.1007965.s005.jpg]
