# Supplementary material for: Global survey of mobile DNA horizontal transfer in arthropods reveals Lepidoptera as a prime hotspot
Source: PLoS Genet. 2019 Feb 1;15(2):e1007965. doi: 10.1371/journal.pgen.1007965 (PMC6373975; doi:10.1371/journal.pgen.1007965)
Supplement: S1 Text — (DOCX) [file pgen.1007965.s014.docx]

## Detailed screening and sequencing results

Highly degenerate primers, overlapping conserved amino acid motifs in arthropod TE proteins, were designed and tested on a reduced sample of 14 specimens from different orders. Four TE superfamilies (out of 11 tested) produced amplicons of the expected size in a wide range of orders and were thus retained for subsequent study: two LTR-retrotransposons (Copia and Gypsy), one non-LTR-retrotransposon (Jockey) and a DNA transposon (Mariner).

The entire sample was screened by PCR for these four superfamilies. Out of the 460 species tested, 365 consistently produced amplicons for at least one of the superfamilies (S1 Table). All amplicons were tagged, mixed and sequenced by 454-pyrosequencing, producing 1,180,083 reads (available in folder 03 of our repository <ftp://pbil.univ-lyon1.fr/pub/datasets/Reiss2019/>). Raw sequences were demultiplexed using the barcode adapters and primer sequences to assign reads to specimens and TE superfamilies, which was achieved for 60% of the reads (S7 Table). High degeneracy of the primers was expected to produce non-specific amplification. In order to retrieve only TE-specific sequences, the demultiplexed reads were blasted against the reference protein sequences initially used for primer design. 81% of the reads carrying a Copia primer, were indeed Copia specific. Similarly, blast confirmed the primer-based family assignation for 60% of the Gypsy reads, 78% of the Jockey reads and 76% of the Mariner reads (S6 Table).

To test for potential contamination, we verified the occurrence of specific TE families in 32 species, using specific PCR and re-sequencing. These spanned 18 TE families, that were recovered in all cases.
