# Supplementary material for: Global survey of mobile DNA horizontal transfer in arthropods reveals Lepidoptera as a prime hotspot
Source: PLoS Genet. 2019 Feb 1;15(2):e1007965. doi: 10.1371/journal.pgen.1007965 (PMC6373975; doi:10.1371/journal.pgen.1007965)

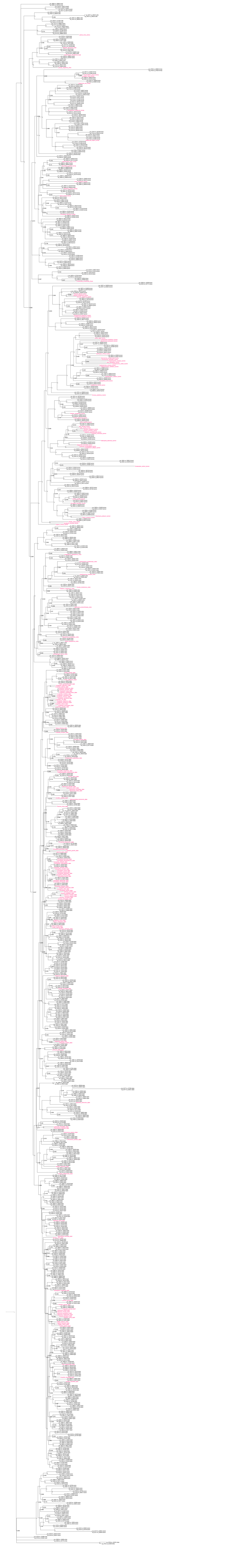

Supplement: S1 Fig — Our specimens are labelled according to the following code: species and specimen ids (cf. S1 Table), followed by 5 letters indicating the order. Species with full genomes are labelled by full species names, followed by 5 letters indicating the order. The tree was computed in RaxML [49] with the mitochondrial gene CO1, using the GTRGAMMA model and constraining the relationships between arthropod orders from the topology of Regier et al [50]. Poorly supported nodes (with SH scores below 0.5) were collapsed in TreeGraph 2 [51]. (PDF) [file pgen.1007965.s001.pdf]
